# Supplementary material for: OTUD6 deubiquitination of RPS7/eS7 on the free 40 S ribosome regulates global protein translation and stress
Source: Nat Commun. 2024 Aug 11;15:6873. doi: 10.1038/s41467-024-51284-y (PMC11316749; doi:10.1038/s41467-024-51284-y)
Supplement: Supplementary file 1 — Supplementary Information [file 41467_2024_51284_MOESM1_ESM.pdf]

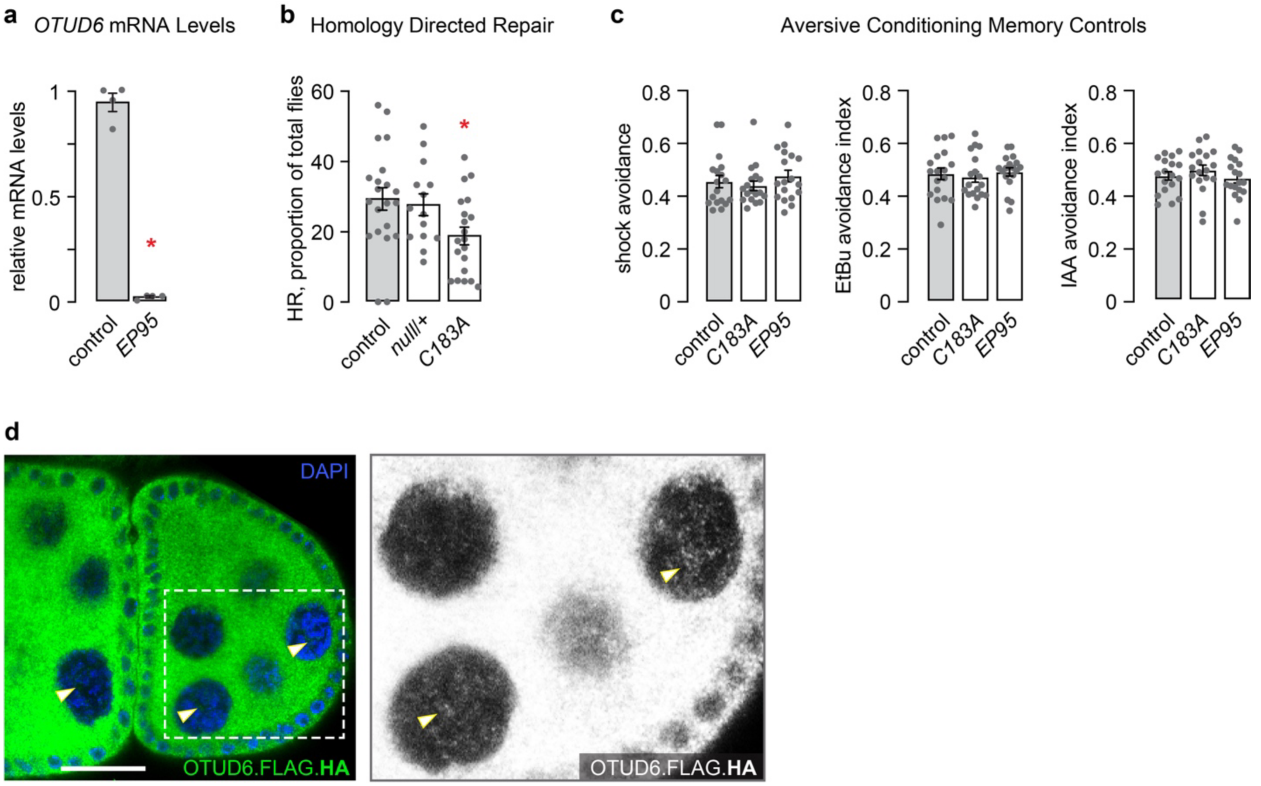

Supplementary Figure 1.

a. Expression levels of OTUD6 in *OTUD6*^{EP95}, measured by qPCR. Two-tailed t-test. (n = 4, 4). **b**. Measurement of the relative amount of double strand break repair events that are by homology-directed repair in *OTUD6* mutants. One-way ANOVA/Dunnett's, compared to control. (n = 21, 15, 20) **c.** Aversive conditioning memory controls: shock avoidance (n = 18, 18, 18), ethyl butyrate avoidance (n = 18, 18, 18), and isoamyl acetate avoidance (n = 18, 18, 18). One-way ANOVA. **d**. OTUD6.FLAG.HA (green) and DAPI (blue) staining of egg chamber nurse cells. Arrowheads point to sites of presumptive nucleolar OTUD6 (HA positive, DAPI negative). Scale bar: 25 µm. Data are presented as mean values +/- SEM. Dots on on bar graphs and n represent biological replicates. Source data and statistics are provided as a Source Data file.

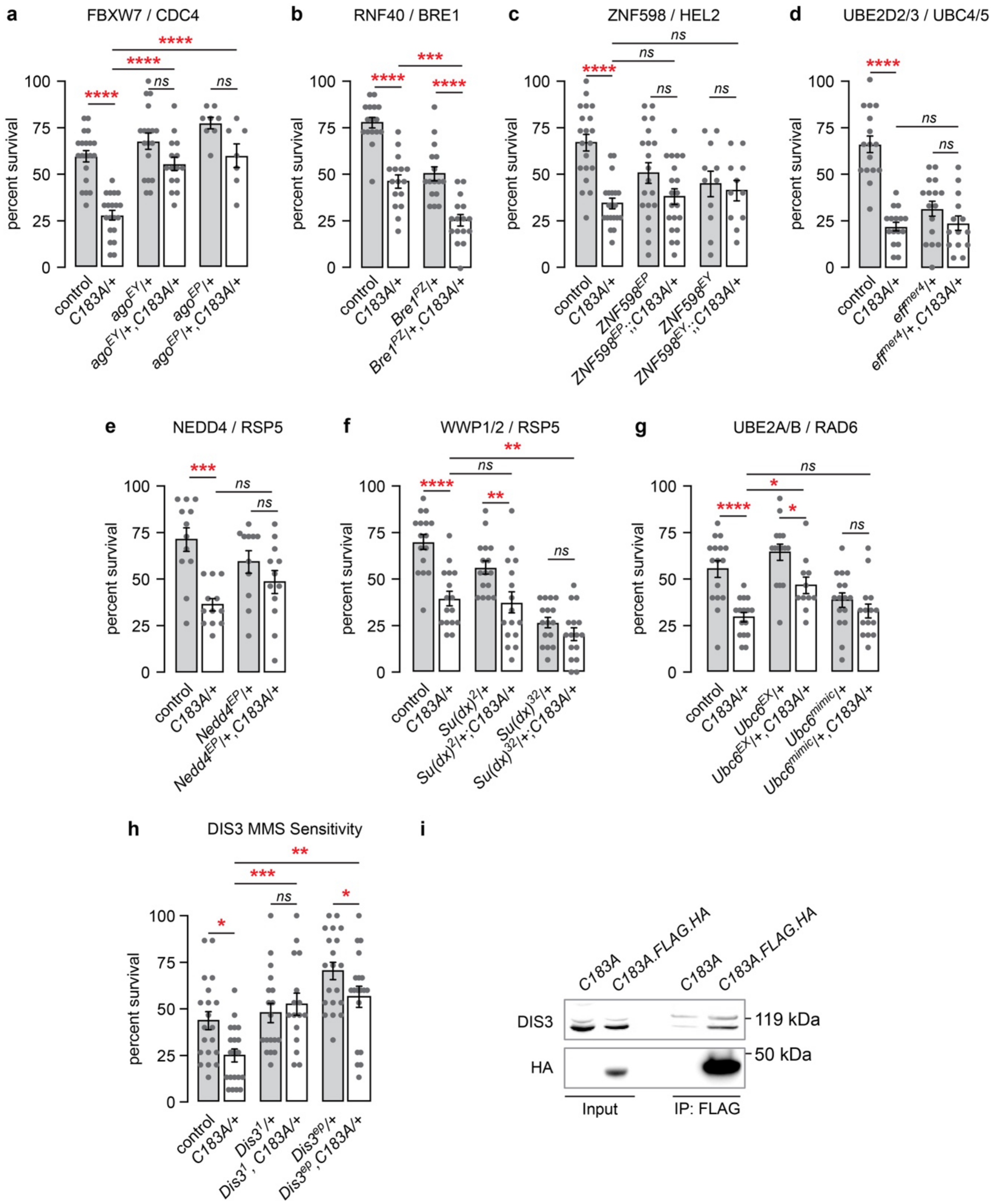

Supplementary Figure 2.

Interactions of catalytically inactive OTUD6 with (**a-g**) E2 conjugating enzymes and E3 ligases, and (**h**) the RNA exosome ribonuclease DIS3, for MMS sensitivity. One way ANOVA with Sidak's multiple comparisons test. (A: n = 20, 20, 18, 15, 8, 7. B: n = 16, 16, 16, 16. C. n = 24, 24, 24, 24, 11, 11. D. n = 16, 16, 16, 14. E: n = 12, 12, 11, 12. F: n = 16, 16, 16, 16, 16, 16. G: n = 16, 16, 15, 11, 16, 15. H: n = 24, 24, 22, 20, 24, 23). **i**. FLAG co-immunoprecipitation with tagged $OTUD6^{C183A}$ followed by western analysis for DIS3 and OTUD6. Data are presented as mean values +/- SEM. Dots on on bar graphs and n represent biological replicates. Source data and statistics are provided as a Source Data file.

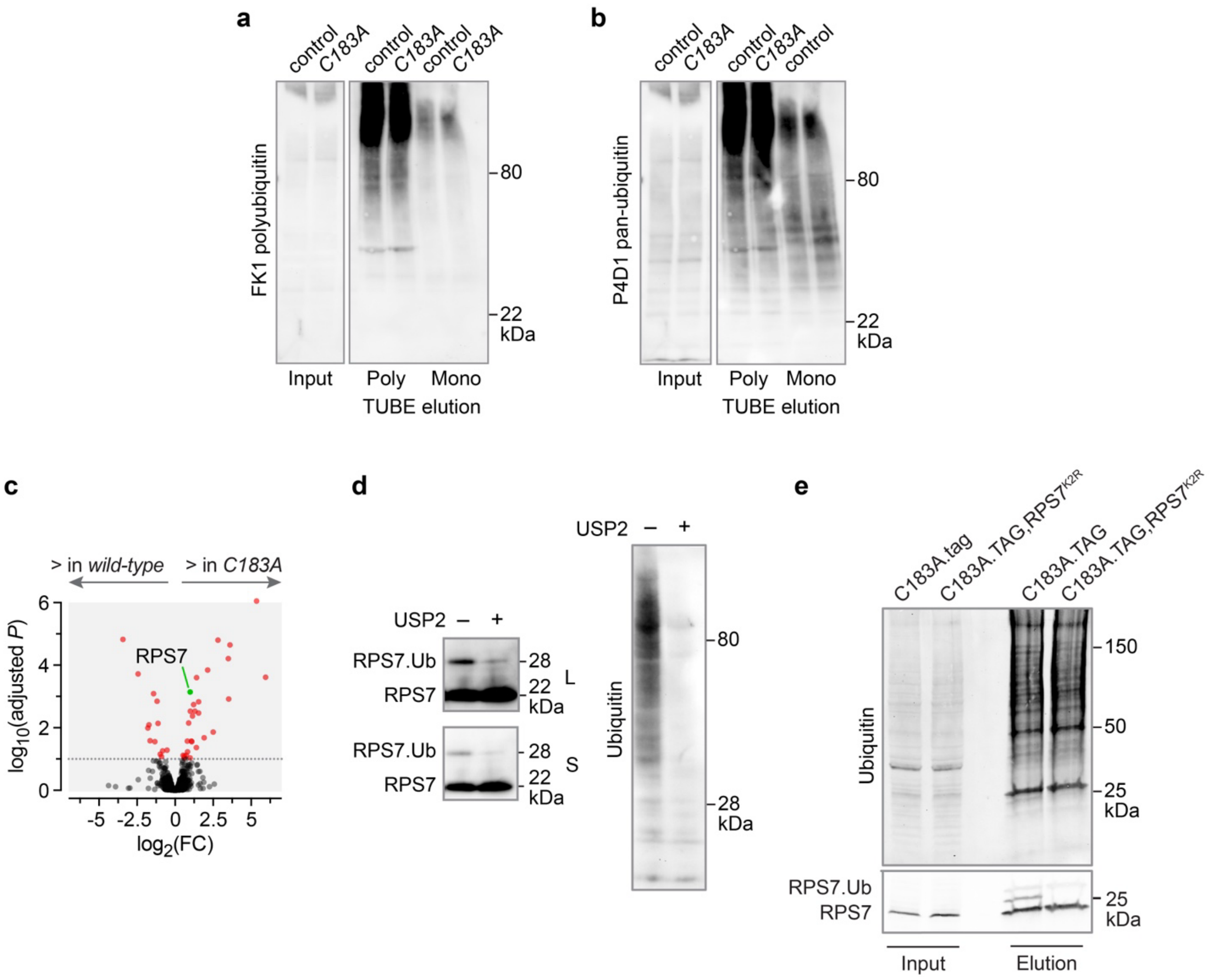

Supplementary Figure 3.

a,**b**. Serial capture of polyubiquitinated then monoubiquitinated proteins enriches for the respective type of ubiquitin chain. **a**: Probed with FK1 antibody that recognizes polyubiquitin chains. **b**: Probed with P4D1 antibody that recognizes all ubiquitin forms. **c**. Mass spectrometry analysis to identify differentially enriched proteins using polyubiquitination-selective capture reagents, between *OTUD6*^{C183A} and the genetic background control. Red indicates significant hits. n = 3 biological replicates per genotype. **d**. Treatment of *OTUD6*^{C183A} head lysate with USP2 deubiquitinase reduces RPS7 monoubiquitination and ubiquitination of most proteins, demonstrating the 28 kDa RPS7 band is due to ubiquitination. **e**. Ubiquitin nanobody capture and RPS7 immunoblotting to detect RPS7.Ub in OTUD6.FLAG.HA with and without RPS7^{K2R}. L: long exposure; S: short exposure. Source data and statistics are provided as a Source Data file.

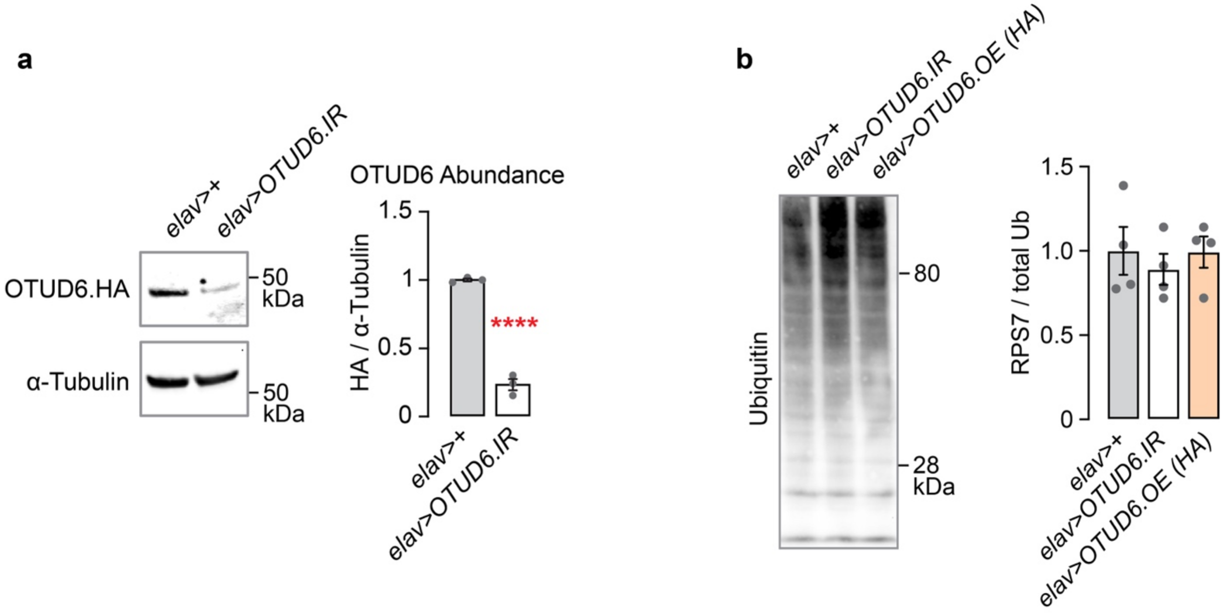

Supplementary Figure 4.

a. Endogenous tagged OTUD6.FLAG.HA protein is markedly reduced in the heads of flies expressing OTUD6 RNAi (*UAS-OTUD6.IR*) in all neurons (*elav-Gal4*). Two-tailed t-test. (n = 3, 3). **b**. Left: overall levels of ubiquitination with underexpression (*elav>OTUD6.IR*) and overexpression (*elav>OTUD6.OE(HA))* in head extracts. Right: levels of nonubiquitinated RPS7 (Fig. 5B) are unaffected by changes in OTUD6 expression. (n = 4, 4, 4). Data are presented as mean values +/- SEM. Dots on on bar graphs and n represent biological replicates. Source data and statistics are provided as a Source Data file.

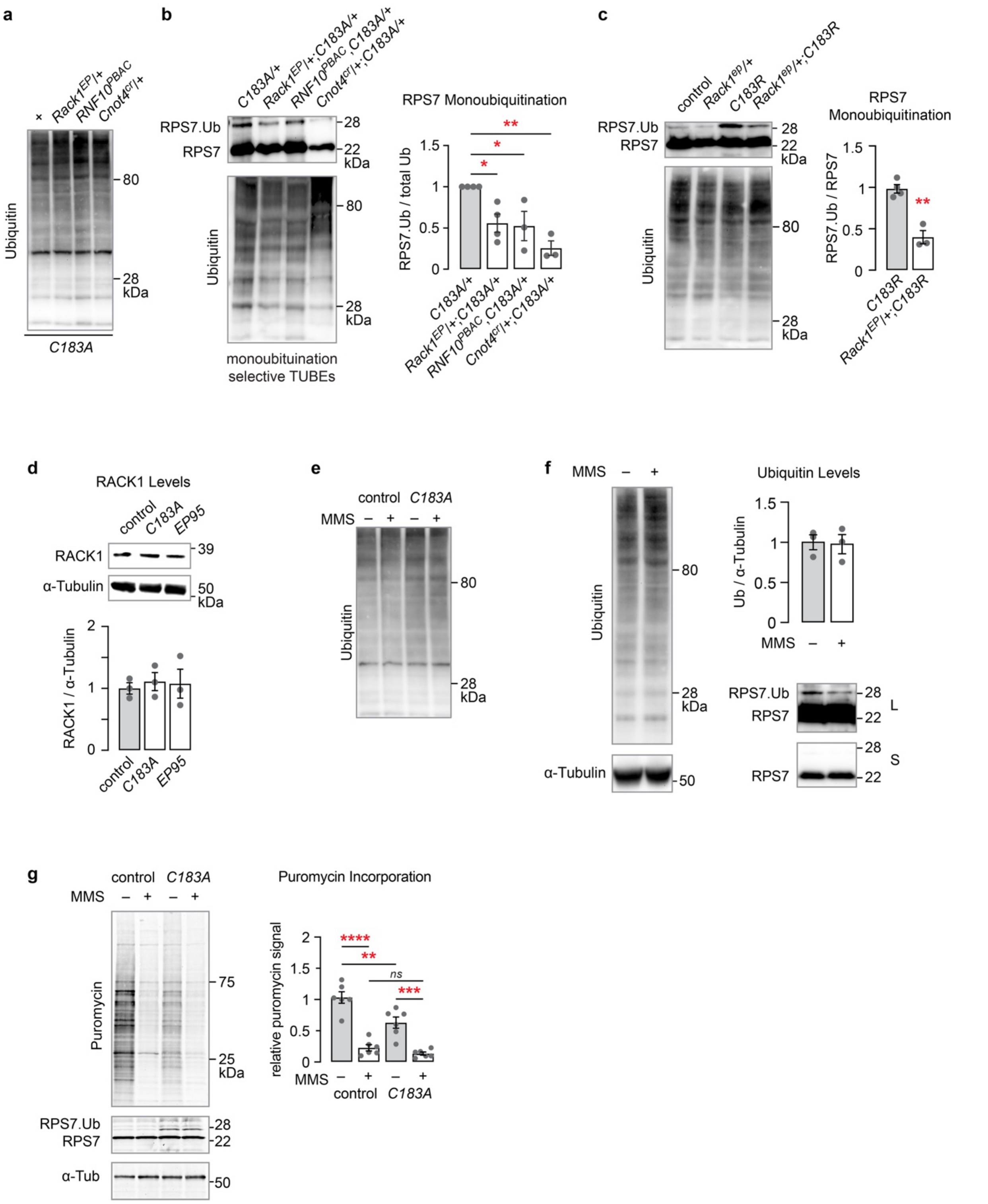

Supplementary Figure 5.

a. Overall ubiquitin signal in flies of the indicated genotypes. Companion to Figure 6A. **b**. RPS7 monoubiquitination levels in fly head extracts purified using monoubiquitination-selective capture reagents from *OTUD6*$^{C183A/+}$ heterozygotes alone and in combination with mutations in *Rack1*, *RNF10*, and *Cnot4*. ANOVA/ Dunnett's compared to control. (n = 4, 4, 3, 3). **c**. RPS7 monoubiquitination in *Rack1 OTUD6*^{C183R}

double mutants. Two-tailed t-test. (n = 3, 3). **d**. RACK1 protein levels were unaffected in OTUD6 catalytically inactive and loss of function mutants. ANOVA. (n = 3, 3, 3). **e**. Overall ubiquitin signal with treatment with MMS. Companion to Figure 6B. **f**. No effect of MMS treatment on total ubiquitin levels. Treatment effectiveness was verified by the observed decrease in RPS7.Ub with MMS treatment. Two-tailed t-test. (n = 3, 3). **g**. MMS decreases global levels of protein translation in control and $OTU6^{C183A}$ flies. One way ANOVA with Sidak's multiple comparisons test. (n = 6, 6, 6, 6). L: long exposure; S: short exposure. Data are presented as mean values +/- SEM. Dots on on bar graphs and n represent biological replicates. Source data and statistics are provided as a Source Data file.

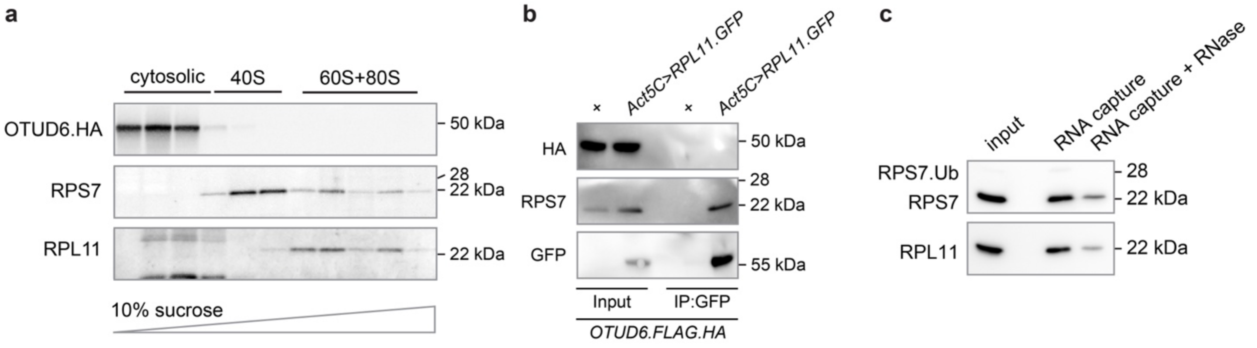

Supplementary Figure 6.

a. Sucrose density fractionation and western detection of tagged OTUD6, RPS7, and RPL11. **b**. Co-IP in the presence of cycloheximide with GFP-tagged RPL11 (*UAS-Rpl11.GFP*) expressed ubiquitously (*Act5C-Gal4*) in OTUD6.FLAG.HA flies, probed with HA, RPS7 and GFP. **c**. RNase treatment of mRNA capture reduces co-capture of RPS7 and RPL11. Source data is provided as a Source Data file.
