# Supplementary material for: OTUD6 deubiquitination of RPS7/eS7 on the free 40 S ribosome regulates global protein translation and stress
Source: Nat Commun. 2024 Aug 11;15:6873. doi: 10.1038/s41467-024-51284-y (PMC11316749; doi:10.1038/s41467-024-51284-y)
Supplement: Supplementary file 2 — Reporting Summary [file 41467_2024_51284_MOESM2_ESM.pdf]

nature portfolio

Corresponding author(s): Fred W Wolf

Last updated by author(s): Jul 15, 2024

Reporting Summary

Nature Portfolio wishes to improve the reproducibility of the work that we publish. This form provides structure for consistency and transparency in reporting. For further information on Nature Portfolio policies, see our Editorial Policies and the Editorial Policy Checklist.

Statistics

For all statistical analyses, confirm that the following items are present in the figure legend, table legend, main text, or Methods section.

n/a	Confirmed	
☐	☒	The exact sample size (*n*) for each experimental group/condition, given as a discrete number and unit of measurement
☐	☒	A statement on whether measurements were taken from distinct samples or whether the same sample was measured repeatedly
☐	☒	The statistical test(s) used AND whether they are one- or two-sided<br>*Only common tests should be described solely by name; describe more complex techniques in the Methods section.*
☐	☒	A description of all covariates tested
☐	☒	A description of any assumptions or corrections, such as tests of normality and adjustment for multiple comparisons
☐	☒	A full description of the statistical parameters including central tendency (e.g. means) or other basic estimates (e.g. regression coefficient) AND variation (e.g. standard deviation) or associated estimates of uncertainty (e.g. confidence intervals)
☐	☒	For null hypothesis testing, the test statistic (e.g. *F*, *t*, *r*) with confidence intervals, effect sizes, degrees of freedom and *P* value noted<br>*Give P values as exact values whenever suitable.*
☒	☐	For Bayesian analysis, information on the choice of priors and Markov chain Monte Carlo settings
☒	☐	For hierarchical and complex designs, identification of the appropriate level for tests and full reporting of outcomes
☒	☐	Estimates of effect sizes (e.g. Cohen's *d*, Pearson's *r*), indicating how they were calculated

Our web collection on statistics for biologists contains articles on many of the points above.

Software and code

Policy information about availability of computer code

Data collection	no software was used
Data analysis	Fiji/ImageJ 2.14.0/1.54f. Adobe Photoshop 2024. Graphpad Prism 10.2.3. MSstatsTMT v2.2.7. Microsoft Excel 16.86.

For manuscripts utilizing custom algorithms or software that are central to the research but not yet described in published literature, software must be made available to editors and reviewers. We strongly encourage code deposition in a community repository (e.g. GitHub). See the Nature Portfolio guidelines for submitting code & software for further information.

Data

Policy information about availability of data

All manuscripts must include a data availability statement. This statement should provide the following information, where applicable:

- Accession codes, unique identifiers, or web links for publicly available datasets
- A description of any restrictions on data availability
- For clinical datasets or third party data, please ensure that the statement adheres to our policy

All data needed to evaluate the conclusions in the paper are present in the Source Data file and in the Supplementary Information file. Raw data from mass spectrometry were deposited within the MassIVE repository under the identifier MSV000091040 (ftp://massive.ucsd.edu/v05/MSV000091040/). Drosophila strains created in this study are deposited at the Bloomington Drosophila Stock Center.

Research involving human participants, their data, or biological material

Policy information about studies with human participants or human data. See also policy information about sex, gender (identity/presentation), and sexual orientation and race, ethnicity and racism.

Reporting on sex and gender	n/a
Reporting on race, ethnicity, or other socially relevant groupings	n/a
Population characteristics	n/a
Recruitment	n/a
Ethics oversight	n/a

Note that full information on the approval of the study protocol must also be provided in the manuscript.

Field-specific reporting

Please select the one below that is the best fit for your research. If you are not sure, read the appropriate sections before making your selection.

☒ Life sciences ☐ Behavioural & social sciences ☐ Ecological, evolutionary & environmental sciences

For a reference copy of the document with all sections, see nature.com/documents/nr-reporting-summary-flat.pdf

Life sciences study design

All studies must disclose on these points even when the disclosure is negative.

Sample size	No statistical methods were used to predetermine sample sizes. Sample sizes are similar to those used in other Drosophila experimental paradigms.
Data exclusions	No data was excluded using statistical methods.
Replication	At least three biological replicates were performed.
Randomization	Samples were grouped according to genotype and treatment. Genetic or treatment controls were included for all experiments.
Blinding	For all experiments where flies were observed the experimenters were blinded to genotype or condition. All other experiments were not blinded.

Reporting for specific materials, systems and methods

We require information from authors about some types of materials, experimental systems and methods used in many studies. Here, indicate whether each material, system or method listed is relevant to your study. If you are not sure if a list item applies to your research, read the appropriate section before selecting a response.

Materials & experimental systems

n/a	Involved in the study	
☐	☒	Antibodies
☒	☐	Eukaryotic cell lines
☒	☐	Palaeontology and archaeology
☐	☒	Animals and other organisms
☒	☐	Clinical data
☒	☐	Dual use research of concern
☒	☐	Plants

Methods

n/a	Involved in the study	
☒	☐	ChIP-seq
☒	☐	Flow cytometry
☒	☐	MRI-based neuroimaging

Antibodies

Antibodies used

Reagent or Resource Source Identifier
Rabbit anti-HA (Western: 1:1,000; IHC: 1:300) Cell Signaling 3724S
Mouse anti-FLAG (1:2,000) Sigma-Aldrich F3165
Rabbit anti-RACK1 (1:5,000) https://doi.org/10.1002/dvdy.21217 N/A

Mouse anti-ubiquitin (P4D1) (1:1,000) Cell Signaling 3936
Mouse anti-Ubiquitinylated proteins,mAb clone FK1 (1:500) Millipore Simga 04-262
Guinea Pig anti-Dis3 (1:5,000) https://doi.org/10.1534/genetics.116.187930 N/A
Rabbit anti-RPL11 (1:1,000) Bethyl Laboratories A303-931A
Chicken anti-RPS7 (1:10,000) This study N/A
Rabbit anti-α-Tubulin (11H10) (1:10,000) Cell Signaling 2125S
Mouse anti-α-Tubulin (1:10,000) Sigma-Aldrich T6074
Mouse anti-Puromycin (1:1,000) Developmental Studies Hybridoma Bank PMY-2A4; AB_2619605
Mouse anti-Bruchpilot (1:20) Developmental Studies Hybridoma Bank NC82; AB_2314866
Rabbit-HRP (1:10,000) Jackson ImmunoResearch 711-035-152; RRID: AB_10015282
Mouse-HRP (1:10,000) Jackson ImmunoResearch 715-035-150; RRID: AB_2340770
Chicken-HRP (1:10,000) Jackson ImmunoResearch 703-035-155; RRID: AB_10015283
Guinea pig-HRP (1:10,000) Invitrogen A18769; RRID: AB_2535546

Validation

Reagent or Resource Validation
Rabbit anti-HA (Western: 1:1,000; IHC: 1:300) Manufacturer's website
Mouse anti-FLAG (1:2,000) Manufacturer's website
Rabbit anti-RACK1 (1:5,000) Original study
Mouse anti-ubiquitin (P4D1) (1:1,000) Manufacturer's website
Mouse anti-Ubiquitinylated proteins,mAb clone FK1 (1:500) Manufacturer's website
Guinea Pig anti-Dis3 (1:5,000) Original study
Rabbit anti-RPL11 (1:1,000) Manufacturer's website, and by molecular weight on Drosophila westerns
Chicken anti-RPS7 (1:10,000) By molecular weight, and by effect of RPS7.K2R mutation
Rabbit anti-α-Tubulin (11H10) (1:10,000) Manufacturer's website
Mouse anti-α-Tubulin (1:10,000) Manufacturer's website
Mouse anti-Puromycin (1:1,000) Manufacturer's website
Mouse anti-Bruchpilot (1:20) Manufacturer's website
Rabbit-HRP (1:10,000) Manufacturer's website
Mouse-HRP (1:10,000) Manufacturer's website
Chicken-HRP (1:10,000) Manufacturer's website
Guinea pig-HRP (1:10,000) Manufacturer's website

Animals and other research organisms

Policy information about studies involving animals; ARRIVE guidelines recommended for reporting animal research, and Sex and Gender in Research

Laboratory animals

Drosophila melanogaster, Berlin genetic strain

Wild animals

n/a

Reporting on sex

Adult males were used for all studies except: 1) olfactory aversive conditioning a 50:50 mix of males and females; 2) Females for collection of ovary egg chambers for immunohistochemistry.

Field-collected samples

n/a

Ethics oversight

not required for invertebrate research

Note that full information on the approval of the study protocol must also be provided in the manuscript.

Plants

Seed stocks

n/a

Novel plant genotypes

n/a

Authentication

n/a
