# Supplementary material for: OTUD6 deubiquitination of RPS7/eS7 on the free 40 S ribosome regulates global protein translation and stress
Source: Nat Commun. 2024 Aug 11;15:6873. doi: 10.1038/s41467-024-51284-y (PMC11316749; doi:10.1038/s41467-024-51284-y)
Supplement: Supplementary file 3 — Description of Additional Supplementary Files [file 41467_2024_51284_MOESM3_ESM.pdf]

Description of Additional Supplementary Files

File Name: Supplementary Data 1
Description: Contains raw data for the OTUD6.C183A.FLAG.HA vs. OTUD6.C183A co-IP mass spectrometry data presented in Figure 2A.

File Name: Supplementary Data 2
Description: Contains raw data for the Polyubiquination Enrichment, control vs. OTUD6.C183A, mass spectrometry data presented in Supplementary Figure 3C.

File Name: Supplementary Data 3
Description: Contains raw data for the Monoubiquination Enrichment, control vs. OTUD6.C183A, mass spectrometry data presented in Figure 4B.

File Name: Supplementary Data 4
Description: Contains reagent descriptions, including *Drosophila* strains, Antibodies, Recombinant DNA, Chemicals Peptides and Recombinant proteins, Software, and Oligonucleotides.
